# Malaria antibody responses augment surveillance in low-transmission settings in the Upper River Region, the Gambia

**DOI:** 10.3389/fepid.2026.1816934

**Published:** 2026-04-21

**Authors:** Rihana A. Aydin, Thomas Keller, Edgard D. Dabira, Nuredin Mohammed, Annette Erhart, Chris Drakeley, Umberto D’Alessandro, Gillian Stresman

**Affiliations:** 1 Department of Epidemiology, College of Public Health, University of South Florida, Tampa, FL, United States; 2 Department of Internal Medicine, Morsani College of Medicine, University of South Florida, Tampa, FL, United States; 3 Disease Control and Elimination Theme, Medical Research Council Unit The Gambia at London School of Hygiene & Tropical Medicine, Fajara, Gambia; 4 Department of Infection Biology, London School of Hygiene & Tropical Medicine, London, United Kingdom

**Keywords:** low-transmission settings, malaria surveillance, mass drug administration, random forest modeling, serological markers

## Abstract

**Introduction:**

In low-transmission malaria settings, routine surveillance often fails to detect asymptomatic infections in partially immune populations. P(Detect), defined as the proportion of infections captured by surveillance systems, serves as a proxy for both population immunity and surveillance completeness. We evaluated whether antibody responses to *Plasmodium falciparum* antigens could classify low vs. high P(Detect) populations and identify surveillance blind spots.

**Methods:**

We analyzed data from 5,300 seropositive individuals across 32 villages in The Gambia following mass drug administration. Random forest classification models were used to predict low vs. high P(Detect) groups based on antibody responses to 19 *P. falciparum* antigens. Analyses were stratified by transmission intensity (<5% and <10% PCR prevalence). Model performance was assessed using precision–recall area under the curve (PR AUC) in held-out test data, and permutation-based variable importance identified key predictive antigens.

**Results:**

Models demonstrated strong discrimination between detectability groups, with PR AUCs of 0.92–0.93 in the <5% stratum and 0.89–0.90 in the <10% stratum using a reduced 8-antigen panel. PfSEA, PfMSP1-19, gSG6, and Etramp4.Ag2 were consistently important predictors, though rankings varied by transmission context. Individual antigen models outperformed combined immune score approaches.

**Discussion:**

Antibody profiles can identify populations where surveillance underestimates transmission due to immunity. Integrating serological data into surveillance systems may improve detection of hidden reservoirs and support more targeted malaria elimination strategies.

## 1 Introduction

Malaria remains a major global health concern, with an estimated 282 million cases reported worldwide in 2024 (1). It is transmitted by *Anopheles* mosquitoes carrying *Plasmodium* parasites, most commonly *P. falciparum* in Africa (1, 2). Despite substantial progress through widespread use of insecticide-treated nets, indoor residual spraying, chemoprevention, and effective antimalarial therapies, countries such as The Gambia continue to face the dual challenge of sustaining control while advancing toward elimination (3, 4).

As transmission declines, an increasing proportion of *P. falciparum* infections become asymptomatic or submicroscopic (below the detection threshold of microscopy and rapid diagnostic tests), escaping routine diagnostics and leading to underestimation of the true infection reservoir (5, 6). Individuals with residual immunity often harbor undetected infections without seeking care, enabling sustained transmission (6, 7). Standard passive surveillance, which depends on symptomatic reporting, therefore, performs poorly in low-endemic settings (5, 7). Although active case detection improves sensitivity, it remains costly and logistically demanding (8, 9). Together, these challenges highlight the need for more sensitive surveillance tools capable of detecting infections that conventional case-based approaches miss.

Serological surveillance can address this gap by capturing both recent and cumulative exposure through antibody responses to specific antigens (10–12). Multiplex bead-based immunoassays enable simultaneous measurement of these responses, facilitating scalable and cost-efficient population-level monitoring (10, 13, 14). Panels spanning parasite life-cycle stages capture distinct dimensions of malaria exposure (Supplementary Table S1). Blood-stage merozoite antigens are the primary targets of naturally acquired immunity and differ in their kinetics — some elicit short-lived responses sensitive to recent or current infection, while others accumulate with repeated exposure and reflect longer-term transmission history (13, 15, 16). Additionally, the *Anopheles* salivary gland protein gSG6 serves as a marker of vector contact intensity independent of confirmed parasite infection (17). While no validated immunological markers exist to directly quantify protection, combining responses across antigens representing these distinct categories improves precision in characterizing exposure history and developing immunity, providing a richer immunological fingerprint than single-antigen approaches (11, 18, 19).

P(Detect) is a metric that estimates the proportion of infections detected by routine surveillance according to transmission intensity, serving as a proxy for population immunity (5). In highly immune populations, infections are more often asymptomatic and undetected, resulting in lower P(Detect) values. This quantitative measure provides a valuable metric for assessing surveillance effectiveness and identifying undetected infection reservoirs, which is particularly critical in settings where rapid transmission reductions have occurred, such as following mass drug administration (MDA) interventions. In these contexts, widespread acquired immunity masks potential resurgence, creating a mismatch between observed clinical cases and actual transmission (20, 21). However, it remains unclear if and how antibody responses reflect changes in population-level immunity and the corresponding implications for surveillance sensitivity.

To address this gap, this study applies random forest modeling to determine whether antibody responses to a panel of *P. falciparum* antigens can predict P(Detect). By linking antibody profiles with detectability across transmission strata, this work identifies immunological markers that reflect the degree to which infections are likely to become symptomatic and thus be detected by routine surveillance, supporting the integration of serology-based metrics into malaria surveillance systems.

## 2 Methods

### 2.1 Data source

This study used data from the MASSIV (Mass Drug Administration of Ivermectin and Dihydroartemisinin-piperaquine as an Additional Intervention for Malaria Elimination) cluster-randomized controlled trial conducted in 32 villages in the Upper River Region of The Gambia between 2018 and 2019 (22, 23). The Upper River Region experiences seasonal malaria transmission that has declined substantially over the past two decades but remains heterogeneous across villages (24). Prior to randomization, 47 villages were screened and those with baseline PCR prevalence below 7% were excluded to ensure sufficient endemicity for the trial. The trial enrolled 10,638 individuals (4,939 intervention; 5,699 control; median age 13 years, 57% female).

Cross-sectional surveys were conducted in November 2018 and November 2019 to assess infection prevalence and collect blood samples for serological analysis. In 2018, malaria prevalence was similar between intervention and control villages, with cluster-level prevalence ranging from 1%–46% and 4%–25%, respectively. By November 2019, prevalence had declined to 5.1% in intervention villages compared with 12.8% in control villages, with cluster-level prevalence ranging from 0%–18% and 5%–51%, respectively.

Villages were randomized to either the intervention or control arm, with a 2-km buffer zone implemented around intervention clusters to minimize spillover effects. Intervention villages received three monthly rounds of MDA each transmission season with ivermectin (300–400 μg/kg/day for three days) and dihydroartemisinin-piperaquine (DP) (either 320/40 mg or 160/20 mg per tablet). Antibody data and PCR prevalence data used to derive P(Detect) were collected during these same survey rounds, ensuring temporal alignment between immune profiles and surveillance detectability metrics. The parent trial demonstrated reductions in clinical malaria incidence, parasite prevalence, and entomological inoculation rates in intervention villages.

### 2.2 P(Detect) (surveillance detectability)

The primary outcome was P(Detect), and values were previously calculated for each village in the MASSIV trial and provided for this analysis (5). P(Detect) estimation integrates PCR-confirmed infection prevalence from cross-sectional surveys with clinical malaria cases detected through routine passive surveillance, accounting for population characteristics and care-seeking behavior. Values range from 0 to 1, with lower values indicating reduced detectability of infections, consistent with settings where more infections are asymptomatic and not captured through routine clinical surveillance. For all individual-level analyses, participants were assigned their village's P(Detect) value. All analyses treated individual observations as independent without explicitly modeling within-village clustering. With 32 villages represented across both transmission strata, this approach provides estimates of the association between antibody profiles and P(Detect) categories.

For random forest classification analyses, individuals were grouped into low vs. high P(Detect) classes based on the P(Detect) value of their village. P(Detect) values were log-transformed to address right skewness, and a two-component Gaussian mixture model was fitted within villages experiencing <10% PCR prevalence. Villages were assigned to classes based on posterior probabilities exceeding 0.5, and this classification was then applied to villages with <5% PCR prevalence to maintain consistent class definitions across prevalence strata. Supplemental random forest regression analyses used continuous P(Detect) values without classification (Supplementary Tables S2, S3).

### 2.3 Antibody and covariate data

Predictors included 19 *P. falciparum* antigens measured using a multiplex bead-based immunoassay (Luminex MAGPIX platform) (22). Serum was eluted from dried blood spot filter paper punches and prediluted to 1:50 in PBS/azide prior to incubation with antigen-coupled beads. Assay plates included BSA-coupled bead controls, malaria naïve negative control sera, and highly exposed Gambian positive control sera, and fluorescence was read on the MAGPIX instrument. Results were expressed as median fluorescence intensity minus background (MFI-bg). The panel was established by the parent MASSIV trial investigators to capture immune responses across multiple *P. falciparum* life-cycle stages—pre-erythrocytic, blood stage merozoite, and mosquito exposure marker—and was selected based on prior evidence of utility in seroepidemiologic surveillance and exposure monitoring (11, 13, 25). Raw MFI-bg values were retained as continuous predictors for model training to preserve interpretability, as random forest algorithms are robust to non-normal distributions. Age (in years) and sex were included in all models as potential confounders. Median antibody levels across all 19 antigens were similar between intervention and control arms in the full study population, and seropositivity rates were nearly identical (70.0% vs. 71.0%); intervention status was therefore not included as a confounder (Supplementary Table S4). Two representations of antibody data were evaluated: (1) separate antigen models with individual antigen responses as distinct predictors, and (2) combined immune score models using a single immune score calculated as the mean MFI across all selected antigens.

### 2.4 Study population and reduced 8 antigen subset

Of the 5,968 individuals available in the parent trial dataset, 5,849 had complete antibody and demographic data. Seropositivity was defined using antigen-specific two-component Gaussian mixture models fit to log-transformed antibody responses [log₁₀(MFI +1)]. Individuals were classified as seropositive if their posterior probability of belonging to the higher-mean component exceeded 0.5 for at least one antigen in the panel. This inclusive definition was used to ensure measurable antibody reactivity within the analytic population, thereby allowing evaluation of relative antigen importance in association with P(Detect), rather than to define recent infection or high-level exposure. This yielded 5,300 seropositive individuals who comprised the primary analytic population.

Primary analyses focused on seropositive individuals because this population represents those with serological evidence of malaria exposure, among whom variation in antibody profiles is most likely to reflect immunologically meaningful differences in exposure history and developing immunity. To assess whether this restriction introduced bias, all analyses were repeated using the full dataset (*n* = 5,849) including seronegative individuals, applying identical outcome definitions, predictor sets, and modeling procedures. Results from the full dataset were consistent with seropositive-only analyses, indicating that our findings are robust to the inclusion criteria (Supplementary Table S2; Supplementary Table S5).

In addition to the full 19-antigen panel, analyses were conducted using a reduced subset of eight antigens to balance model simplicity and interpretability with predictive performance. To identify this subset, we implemented an iterative variable ordering approach using random forest regression models predicting continuous P(Detect). Antigens were ranked based on their cumulative contribution to model performance, and models were sequentially refit with increasing numbers of predictors. Model performance was evaluated using out-of-sample R^2^. Visual inspection of the cumulative R^2^ curves across both transmission strata showed that performance gains diminished and became unstable after approximately eight antigens, with subsequent additions yielding no consistent improvement (Supplementary Figure S1). The following eight antigens were therefore selected to balance model simplicity and interpretability with predictive performance: gSG6, Etramp4.Ag2, SBP1, Rh5.1, Rh4.2, PfSEA, MSP2.Dd2, and PfMSP1-19.

### 2.5 Statistical analysis

Random forest classification models were used to determine whether a set of antibody measurements could reliably distinguish individuals living in villages where malaria surveillance performs poorly [low P(Detect)] from those where surveillance performs adequately [high P(Detect)]. Random forests are ensemble machine learning methods that build multiple decision trees on random subsets of the data and aggregate their predictions; they are well-suited to this study as they capture non-linear relationships and are robust to correlation among predictors, both of which are properties of multiplex antibody data (19). Model performance was evaluated on data withheld during training (held out test set), ensuring the reported performance metrics reflect generalizability rather than overfitting.

All analyses were conducted in R version 4.3.1 using the tidymodels framework (26, 27). Models were implemented with the parsnip package using the ranger engine (28). Additional packages included mclust for mixture model classification, yardstick for performance metrics, vip for permutation-based variable importance, and dplyr and ggplot2 for data visualization (29–33). Analyses were stratified by transmission intensity (<5% and <10%) to evaluate whether antibody-detectability relationships differed across epidemiological contexts.

Within each transmission stratum, data were randomly split into training (80%) and testing (20%) sets using rsample, with stratification by P(Detect) class (34). Hyperparameter tuning was performed using 10-fold cross-validation via the tune package, with final values selected based on optimal performance in the validation folds (35). Specifically, the number of variables randomly sampled at each split (mtry) and the minimum number of observations required in a terminal node (min_n) were jointly optimized. For the all-19 antigen models, optimal hyperparameters were mtry = 2 and min_*n* = 8 in the <10% transmission stratum and mtry = 3 and min_*n* = 48 in the <5% stratum. For the reduced 8-antigen panel models, optimal values were mtry = 2 and min_*n* = 8 in both transmission strata. All random forest models used 500 trees.

Because individuals living in low P(Detect) villages comprised approximately 73%–82% of each stratum, standard accuracy metrics can be misleading—a model predicting the majority class for all individuals would achieve high accuracy while proving no useful discrimination. Therefore, precision-recall area under the curve (PR AUC) was used as the primary performance metric (30). PR AUC summarizes a model's ability to correctly identify individuals in low-detectability settings across all possible classification thresholds while minimizing false positive classifications; values approaching 1.0 indicate strong discrimination between low and high detectability groups. Receiver operating characteristic area under the curve (ROC AUC) and overall accuracy were also reported for completeness (30). Low P(Detect) was treated as the positive class. The same framework, data splitting strategy, and hyperparameter tuning procedures were applied to the supplemental random forest regression analyses, for which model performance was assessed using root mean squared error (RMSE) and R^2^ (30) (Supplementary Tables S2, S3).

To aid interpretation of the antibody data and explore potential demographic patterns, additional descriptive visualizations were generated among seropositive individuals in the primary analytic population. Scatterplots were generated showing MFI-bg responses for each antigen in the reduced 8-antigen panel plotted against continuous village-level P(Detect) (Supplementary Figure S2). Violin plots were used to visualize the distribution of MFI-bg responses for the reduced 8-antigen panel stratified by age group and by sex (Supplementary Figures S3, S4). Age was categorized into five groups (0–5, 6–15, 16–30, 31–45, and ≥46 years) for visualization purposes only; age was retained as a continuous variable in all models.

To complement the random forest classification results and assess whether associations between individual antibody responses and P(Detect) were detectable using a simpler, more interpretable approach, linear regression models were fit examining associations between standardized MFI-bg responses and continuous P(Detect) for each antigen in the reduced 8-antigen panel, both unadjusted and adjusted for age and sex. These analyses were conducted among seropositive individuals and results are reported in Supplementary Table S6.

## 3 Results

Study populations were comparable across transmission strata, with similar sex distributions and age ranges (Table 1). Median P(Detect) was higher in areas with >10% prevalence (0.17) than in <10% prevalence areas (0.12), consistent with greater detectability of infections in higher-transmission settings. Median antibody responses were generally higher in the higher-prevalence stratum, with GLURP.R2 and PfAMA1 showing the most pronounced differences, although neither consistently ranked among the strongest predictors in multivariable models.

**Table 1:** Baseline characteristics of seropositive study population stratified by transmission level.

Variable	<5%	>5%	<10%	>10%
Total participants (n)	1,922	3,378	4,075	1,225
Sex, female (*n*%)	60.0	56.8	59.1	54.2
Age, years	15 (0–103)	13 (0–104)	14 (0–104)	13 (0–98)
P(Detect)	0.13 (0.07–0.65)	0.12 (0.02–0.55)	0.12 (0.04–0.65)	0.17 (0.02–0.38)
Antibody responses, MFI-bg				
gSG6	645 (82–26,399)	722 (82–18,200)	675 (82–26,399)	756 (94–17,359)
Etramp4.Ag2	1,344 (212–37,084)	1,750 (128–36,366)	1,483 (128–37,084)	2,017 (129–36,366)
SBP1	1,038 (166–12,675)	1,184 (98–17,268)	1,078 (98–16,050)	1,290 (142–17,268)
Rh5.1	1,574 (83–9,990)	1,826 (90–9,216)	1,690 (83–9,990)	1,877 (90–8,906)
Rh4.2	1,009 (91–33,015)	1,252 (92–31,237)	1,106 (91–33,015)	1,384 (107–31,237)
PfSEA	1,372 (179–24,154)	1,502 (165–24,350)	1,372 (165–24,350)	1,790 (245–21,323)
MSP2.Dd2	1,536 (124–27,997)	1,732 (3–28,531)	1,606 (124–28,531)	1,915 (3–23,641)
PfMSP1-19	3,428 (141–36,894)	5,294 (146–36,634)	3,933 (141–36,894)	7,175 (165–36,634)
Etramp5.Ag1	802 (88–37,744)	1,010 (82–36,554)	870 (82–37,744)	1,216 (104–36,035)
EBA181.RIII-V	830 (78–32,874)	924 (106–27,477)	845 (78–32,874)	1,130 (196–27,477)
Hyp2	1,551 (264–27,051)	1,678 (242–22,071)	1,586 (242–27,051)	1,831 (294–22,071)
CSP	568 (55–29,717)	896 (47–32,540)	663 (47–32,540)	1,264 (50–30,335)
EBA140.RIII-V	454 (40–40,436)	693 (42–38,685)	544 (40–40,436)	826 (44–38,685)
EBA175.RIII-V	400 (32–47,768)	710 (34–43,295)	484 (32–47,768)	1,128 (42–43,278)
GLURP.R2	1,674 (37–55,930)	3,080 (38–54,998)	1,874 (37–55,930)	6,541 (43–54,547)
HSP40.Ag1	1,335 (188–21,872)	1,618 (132–30,473)	1,412 (132–30,473)	1,842 (149–28,667)
MSP2.CH150	1,102 (81–38,080)	1,957 (75–41,043)	1,354 (77–38,080)	2,920 (75–41,043)
PfAMA1	4,331 (87–35,608)	7,014 (82–38,976)	4,928 (82–35,608)	8,244 (98–38,976)
Rh2.2030	1,542 (193–35,932)	2,265 (158–35,120)	1,542 (193–35,932)	2,942 (261–35,120)

All continuous variables are reported as median (minimum, maximum). P(Detect) represents the modeled proportion of *P. falciparum* infections detected by routine surveillance. Antigen-specific IgG responses are reported as raw median fluorescence intensity minus background (MFI-bg).

Scatterplots of MFI-bg responses for each antigen in the reduced 8-antigen panel against continuous village-level P(Detect) showed weak linear trends for several markers, while others exhibited minimal monotonic association, suggesting that predictive performance arises from multivariate combinations of antibody signals rather than strong individual-marker effects (Supplementary Figure S2). Violin plots stratified by age group showed age-dependent increases in antibody responses for several antigens—particularly blood-stage markers including PfMSP1-19, Rh5.1, and PfSEA—consistent with patterns of cumulative exposure through repeated infection (Supplementary Figure S3). Antibody distributions were broadly similar between males and females across the reduced 8-antigen panel, with no consistent or biologically meaningful sex-specific differences observed (Supplementary Figure S4).

**Figure 1:**
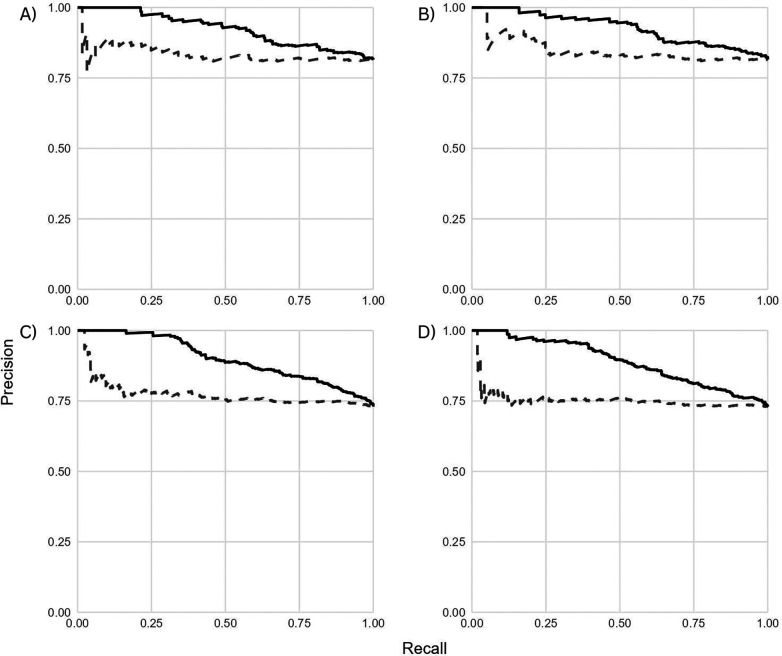
Precision-recall curves for classification models predicting low vs. high P(Detect) among seropositive individuals. Models using individual antigen responses as separate predictors (solid line) outperformed models using combined mean MFI immune scores (dashed line) across both transmission strata. **(A)** <5% transmission, all 19 antigens combined; **(B)** <5% transmission, reduced 8 antigens combined; **(C)** <10% transmission, all 19 antigens combined; **(D)** <10% transmission, reduced 8 antigens combined. PR AUC values indicate model precision in identifying individuals with low detectability.

Classification models using individual antigens as separate predictors achieved strong performance in distinguishing low vs. high detectability groups across both transmission settings (Table 2). In the <5% stratum, the reduced 8-antigen model achieved a PR AUC of 0.93 and an accuracy of 0.83, outperforming combined mean MFI models (PR AUC = 0.83). Similar patterns were observed in the <10% stratum, where separate-antigen models achieved PR AUC values of 0.89–0.90 compared with 0.76–0.77 for combined-antigen approaches (Table 2; Figure 1).

**Table 2:** Performance metrics of random forest classification models predicting low vs. high P(Detect) in seropositive individuals across transmission settings.

Transmission Level	Model Type	Antigen Set	ROC AUC	PR AUC	Accuracy
<5%	Separate	All 19	0.72	0.92	0.82
		Reduced 8	0.73	0.93	0.83
	Combined mean MFI	All 19	0.58	0.85	0.81
		Reduced 8	0.53	0.83	0.78
<10%	Separate	All 19	0.76	0.90	0.75
		Reduced 8	0.73	0.89	0.74
	Combined Mean MFI	All 19	0.55	0.77	0.69
		Reduced 8	0.52	0.76	0.68

Panel designations (A–D) referenced in the figure captions correspond to the position of panels within each figure (e.g., top left to bottom right).

**Figure 2:**
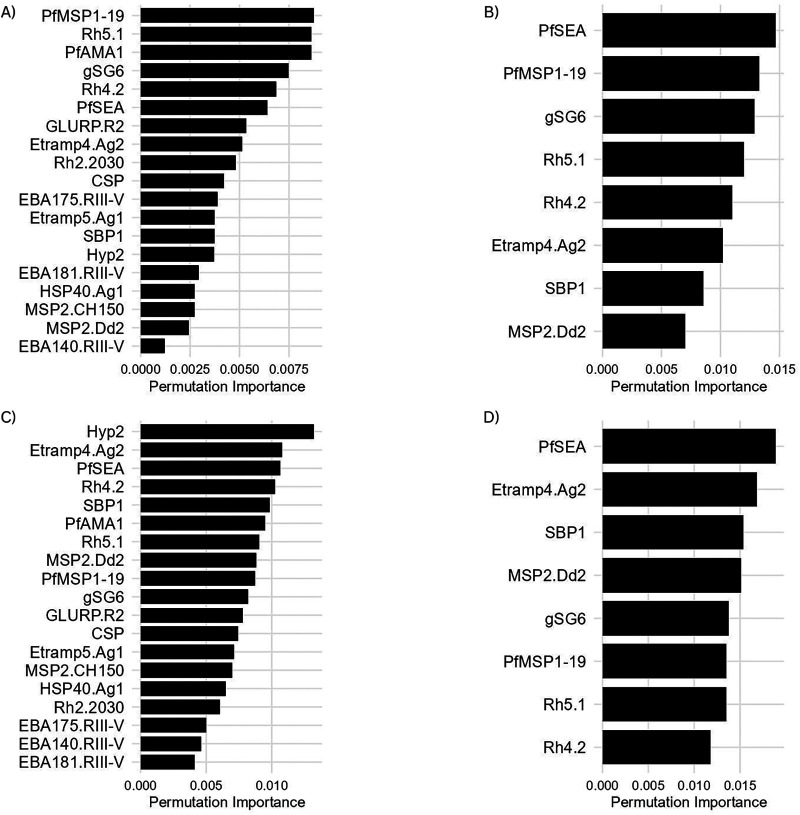
Variable importance of antibody predictors from random forest classification models across transmission settings among seropositive individuals. **(A)** <5% transmission, all 19 antigens; **(B)** <5% transmission, reduced 8 antigens. **(C)** <10% transmission, all 19 antigens. **(D)** <10% transmission, reduced 8 antigens. Higher importance values indicate greater influence on classification performance.

Variable importance analyses identified distinct sets of antigens associated with P(Detect) classification across transmission strata (Figure 2). In the <5% PCR prevalence stratum using all 19 antigens, the strongest predictors were PfMSP1-19, Rh5.1, and PfAMA1 (Figure 2A). When the analysis was restricted to the reduced 8-antigen panel in this stratum, PfSEA ranked as the most important predictor, followed by PfMSP1-19 and gSG6 (Figure 2B). In the <10% transmission stratum, a different pattern was observed: in the full 19-antigen model, Hyp2 and Etramp4.Ag2 ranked highest in permutation importance (Figure 2C), while PfSEA remained the strongest predictor in the reduced 8-antigen model, followed by Etramp4.Ag2 and SBP1 (Figure 2D). Antigens associated with cumulative exposure, including PfAMA1 and GLURP.R2, ranked lower in importance in the <10% stratum across both antigen sets. Age and sex were included as covariates in all models but consistently ranked lowest in permutation importance across all four models, indicating that demographic factors contributed minimally to P(Detect) classification relative to antibody responses (Supplementary Figures S3, S4).

Analyses including the full dataset with seronegative individuals (*n* = 5,849) produced results consistent with those from the seropositive-only analyses, with comparable model performance and variable importance patterns (Supplementary Tables S2, S3). MSP2.Dd2 increased in relative importance when seronegative individuals were included. Results from supplemental random forest regression models using continuous P(Detect) as the outcome are provided in Supplementary Figure S5 and Supplementary Table S3. In complementary linear regression analyses among seropositive individuals, several antigens identified as important predictors in the random forest models—including gSG6, Etramp4.Ag2, Rh5.1, and MSP2.Dd2—were also significantly associated with continuous P(Detect) after adjustment for age and sex, providing convergent evidence that these markers capture meaningful variation in surveillance detectability using a simpler analytical approach. Other markers showed weaker or non-significant linear associations, consistent with the interpretation that some predictive signal in the random forest models reflects non-linear or interaction effects not captured by linear regression (Supplementary Table S6).

## 4 Discussion

This study demonstrates that antibody profiles capturing both recent parasite exposure and cumulative immune responses can identify populations where routine malaria surveillance fails to detect ongoing transmission. Across both transmission strata (<5% and <10% PCR prevalence), random forest classification models achieved strong discrimination between populations with low vs. high detectability (PR AUC ≈0.9), indicating that antibody panels can flag areas where infections are missed by passive surveillance. In contrast, regression models predicting continuous P(Detect) performed modestly (R^2^ < 0.15), suggesting that antibody responses distinguish broad categories of surveillance performance but capture only a portion of the continuous variation in detectability. Together, these findings indicate that serological data can serve as indicators of surveillance system completeness, helping programs identify populations where infections may be systematically missed. Although the multiplex assay used here requires specialized laboratory infrastructure, the strong performance of the reduced 8-antigen panel suggests that fewer markers may retain substantial discriminatory value, supporting translation into simpler platforms such as ELISA-based assays or antigen-specific rapid diagnostic tests for integration into malaria surveillance programs.

The antigens identified as the strongest predictors represent immune responses to distinct biological processes that help explain why some infections escape routine surveillance. Markers associated with recent parasite exposure (e.g., PfSEA, Etramp4.Ag2, Etramp5.Ag1, MSP2.Dd2, and Hyp2) featured prominently in the <10% transmission stratum, suggesting that ongoing parasite activity shapes detectability at moderate-low transmission. In contrast, long-lived markers of cumulative exposure (e.g., PfMSP1-19, Rh5.1, and PfAMA1) were more prominent in the <5% stratum, indicating that historically acquired immunity may play a larger role in determining whether infections remain asymptomatic and therefore undetected in very low transmission settings. The recurrent importance of gSG6, a mosquito salivary gland protein reflecting vector contact rather than infection, further suggests that greater mosquito exposure—and thus repeated parasite inoculation—may promote immune profiles associated with asymptomatic carriage. Together, these findings indicate that surveillance detectability likely reflects the combined influence of recent parasite exposure, cumulative immunity, and vector contact.

The stronger performance of antigen-specific models compared with composite mean MFI approaches likely reflects information loss when distinct immunological signals are collapsed into a single summary measure. Individual antigens capture different aspects of malaria exposure and transmission dynamics. For example, Etramp5.Ag1 reflects recent parasite exposure due to its short antibody half-life, gSG6 indicates vector contact, and blood-stage antigens such as PfMSP1-19 and PfAMA1 represent cumulative exposure. Averaging these signals can obscure meaningful immunological patterns; individuals with high recent exposure but limited cumulative immunity may have similar mean MFI values to those with long-term exposure but little recent transmission despite representing distinct epidemiological profiles. PfAMA1 and GLURP.R2—both markers of cumulative exposure—did not rank among the strongest predictors in the reduced panel, likely reflecting redundancy among correlated blood-stage antigens. These results suggest that antigen-specific antibody profiles may provide more informative surveillance signals than aggregate immune scores. The superior performance of classification compared with regression models likely reflects both the operational utility of categorical surveillance metrics and inherent limitations in estimating continuous P(Detect). Distinguishing populations with poor vs. adequate surveillance sensitivity provides more actionable information for program decision-making than precise estimation of detectability values. In addition, both predictor and outcome variables contain substantial measurement variability. Antibody levels fluctuate with immune responses and exposure timing, while P(Detect) itself is a modeled estimate subject to uncertainty in infection prevalence, healthcare-seeking behavior, and model assumptions. Classification models, which divide observations into discrete surveillance performance categories, are therefore more robust to this variability and better suited to capture broad differences in detectability.

These findings build on growing evidence that antibody data can improve malaria surveillance in low-transmission and elimination settings (10, 11, 13). As transmission declines, asymptomatic infections increasingly escape routine passive surveillance, causing reported case numbers to underestimate true transmission intensity and mask hidden reservoirs (5). By linking multiplex antibody profiles with modeled P(Detect), this study demonstrates that serology can contextualize routine case data and identify populations with disproportionately low detectability that may benefit from targeted interventions such as active case detection, focal screen-and-treat campaigns, or intensified vector control. Importantly, this study quantitatively connects immune responses to a modeled surveillance metric through a machine learning framework, bridging immuno-epidemiological patterns with operational measures of surveillance effectiveness.

Several limitations should be acknowledged. First, although classification models achieved high performance, they were trained and tested within a single geographic region following mass drug administration. Internal validation demonstrated strong performance, but models have not been validated in other populations with differing transmission histories, vector species, or parasite diversity. Second, antibody persistence means that many immune profiles reflect both recent and historical exposure, which may limit the ability to distinguish populations with genuinely ongoing transmission from those with recent but now-interrupted transmission, particularly in post-intervention settings, although some antigens are more strongly associated with short-term exposure (11). Third, seropositivity was defined as reactivity to at least one antigen to ensure inclusion of individuals with measurable antibody responses for evaluating relative antigen importance in relation to P(Detect). More conservative definitions of seropositivity may preferentially capture individuals with repeated or recent exposure and could yield different patterns of variable importance. Fourth, the modest R^2^ values from regression analyses indicate that unmeasured factors such as vector behavior, microgeographic transmission heterogeneity, and temporal variation in healthcare-seeking likely contribute to continuous detectability variation. Fifth, P(Detect) is a village-level metric, but our analyses treated individual observations as independent without explicitly modeling within-village correlation. Consequently, our confidence intervals and performance metrics may be somewhat optimistic. Nevertheless, the strong and consistent performance observed across both transmission strata and across different antigen panels suggests robust discrimination despite this limitation.

Future research should prioritize validation across diverse epidemiological settings and transmission contexts, with attention to analytical frameworks that account for clustering through hierarchical modeling or village-level aggregation. Longitudinal applications of antibody-based detectability modeling could track changes in surveillance performance following interventions or seasonal fluctuations. Standardized antigen panels validated across regions will be essential for cross-program comparability and implementation. Integrating serology with routine surveillance and entomological monitoring could provide a more comprehensive picture of hidden transmission and improve predictive accuracy. Embedding antibody-based surveillance indicators into digital health platforms or decision-support systems could operationalize their use for real-time program management and resource allocation.

Overall, this study establishes that antibody profiles can serve as practical indicators of surveillance system performance in low-transmission settings, quantitatively linking immune responses to the degree to which routine surveillance captures ongoing transmission. By identifying populations where case data systematically underestimates infection burden due to population immunity, serological surveillance provides programs with a scalable tool to reveal hidden transmission and target enhanced surveillance where it is most needed.

## Data Availability

The raw data supporting the conclusions of this article will be made available by the authors, without undue reservation.
