# Supplementary material for: Malaria antibody responses augment surveillance in low-transmission settings in the Upper River Region, the Gambia
Source: Front Epidemiol. 2026 Apr 21;6:1816934. doi: 10.3389/fepid.2026.1816934 (PMC13139072; doi:10.3389/fepid.2026.1816934)
Supplement: Supplementary file 1 [file Table1.docx]

Supplementary Material

# Supplementary Figures

## *Note: Panel designations (A–D) referenced in the figure captions correspond to the position of panels within each figure (e.g., top left to bottom right for 2×2 layouts and top to bottom for vertically stacked panels).*

## Supplementary Figure 1. Identification of top antigens based on cumulative model performance.


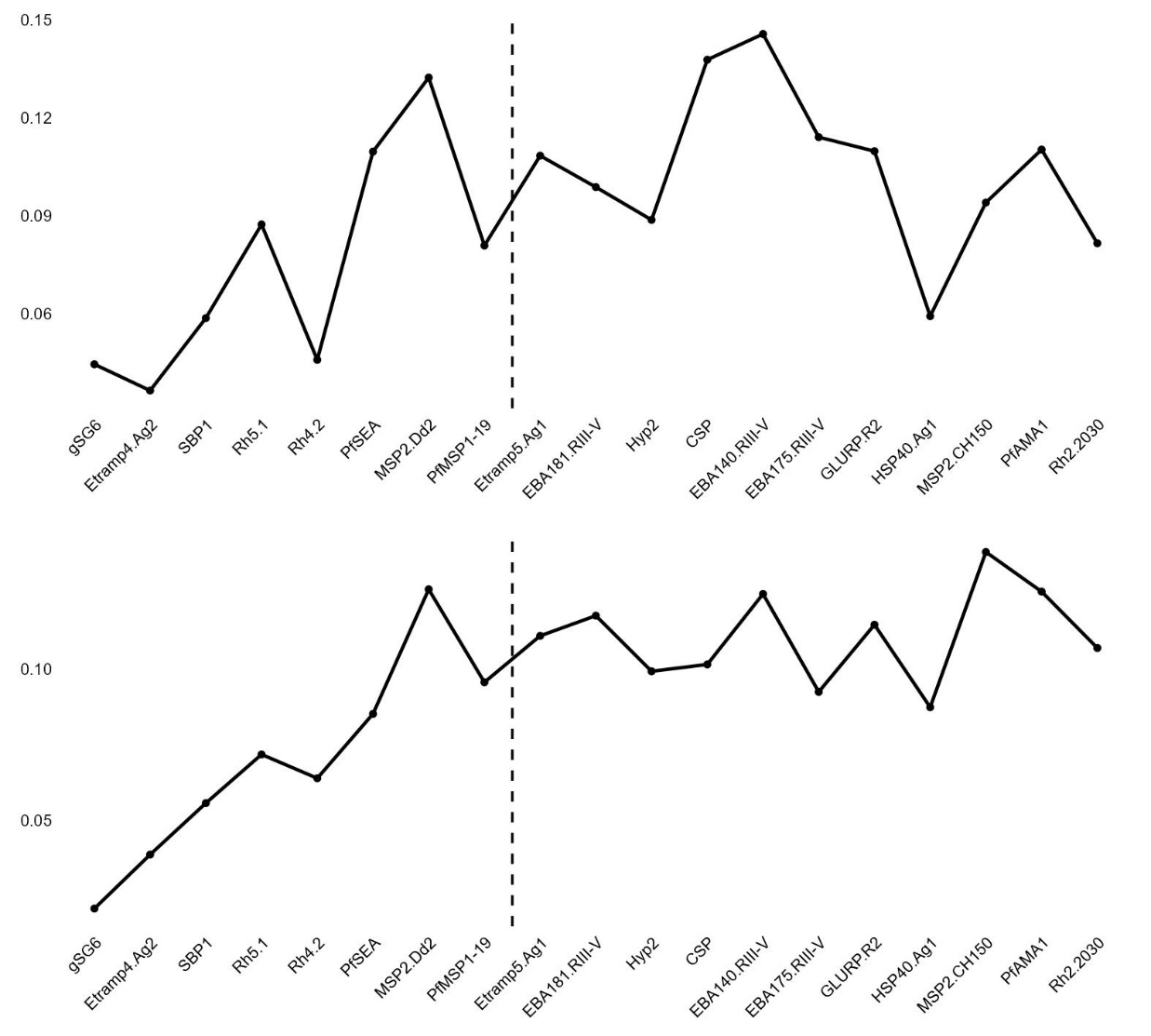


**(A)** cumulative model R² for <5% transmission; **(B)** cumulative model R² for <10% transmission. The black vertical dashed line marks the point of model stabilization used to define the reduced 8-antigen panel.

**Supplementary Figure 2.** Antibody responses to top 8 predictive antigens across continuous P(Detect)

**
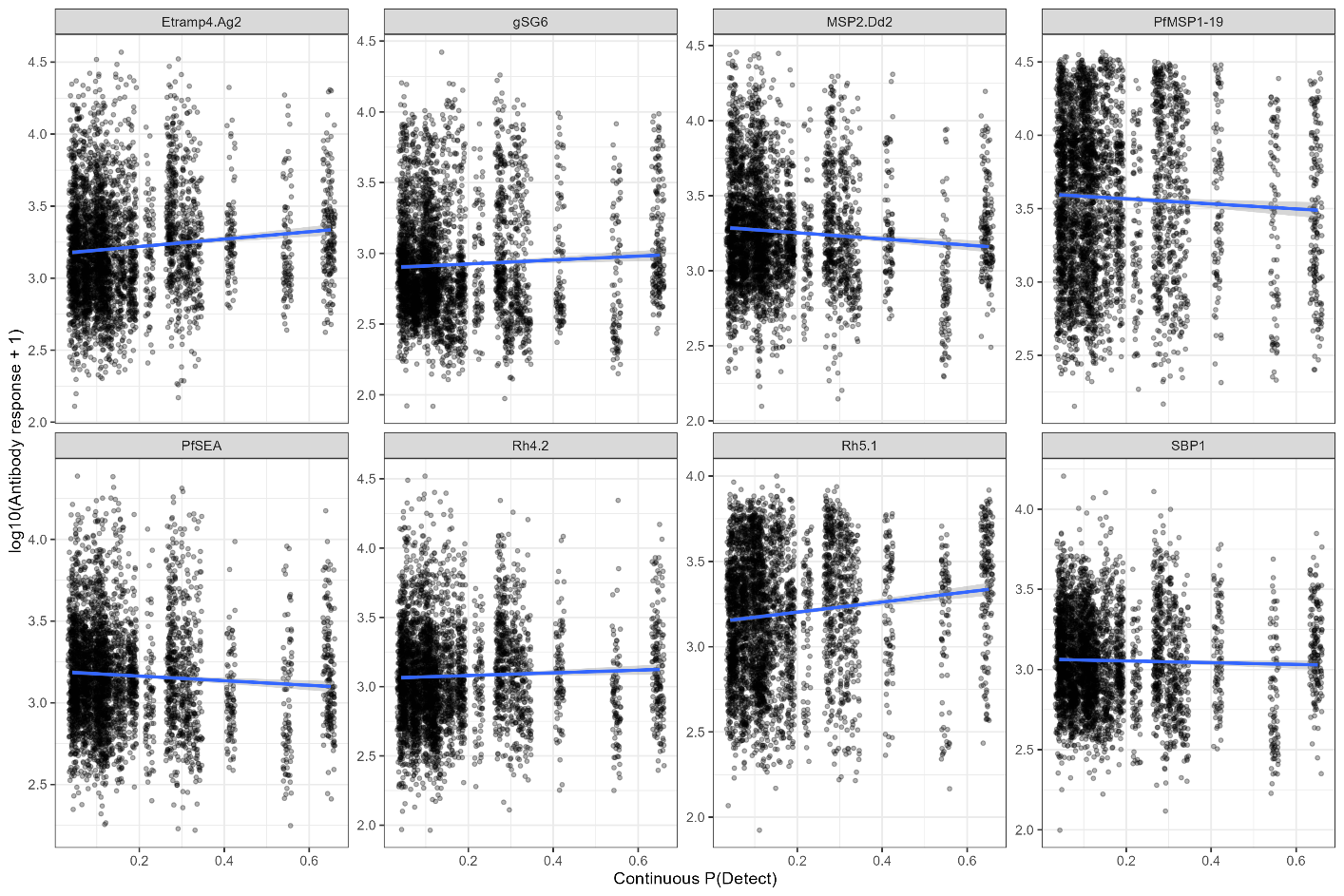
**

Scatterplots showing the relationship between antibody responses and continuous P(Detect) among seropositive individuals in the <10% transmission stratum. Each point represents an individual participant. The x-axis shows the modeled surveillance detectability metric, P(Detect), while the y-axis shows log10-transformed antibody responses (MFI + 1). Panels display the 8 antigens with the highest importance in the random forest classification models. Blue lines represent fitted linear trends summarizing the overall association between antibody levels and P(Detect). Vertical clustering of points reflects shared village-level P(Detect) values among participants.

**Supplementary Figure 3.** Age-specific distribution of antibody responses to top 8 predictive antigens

**
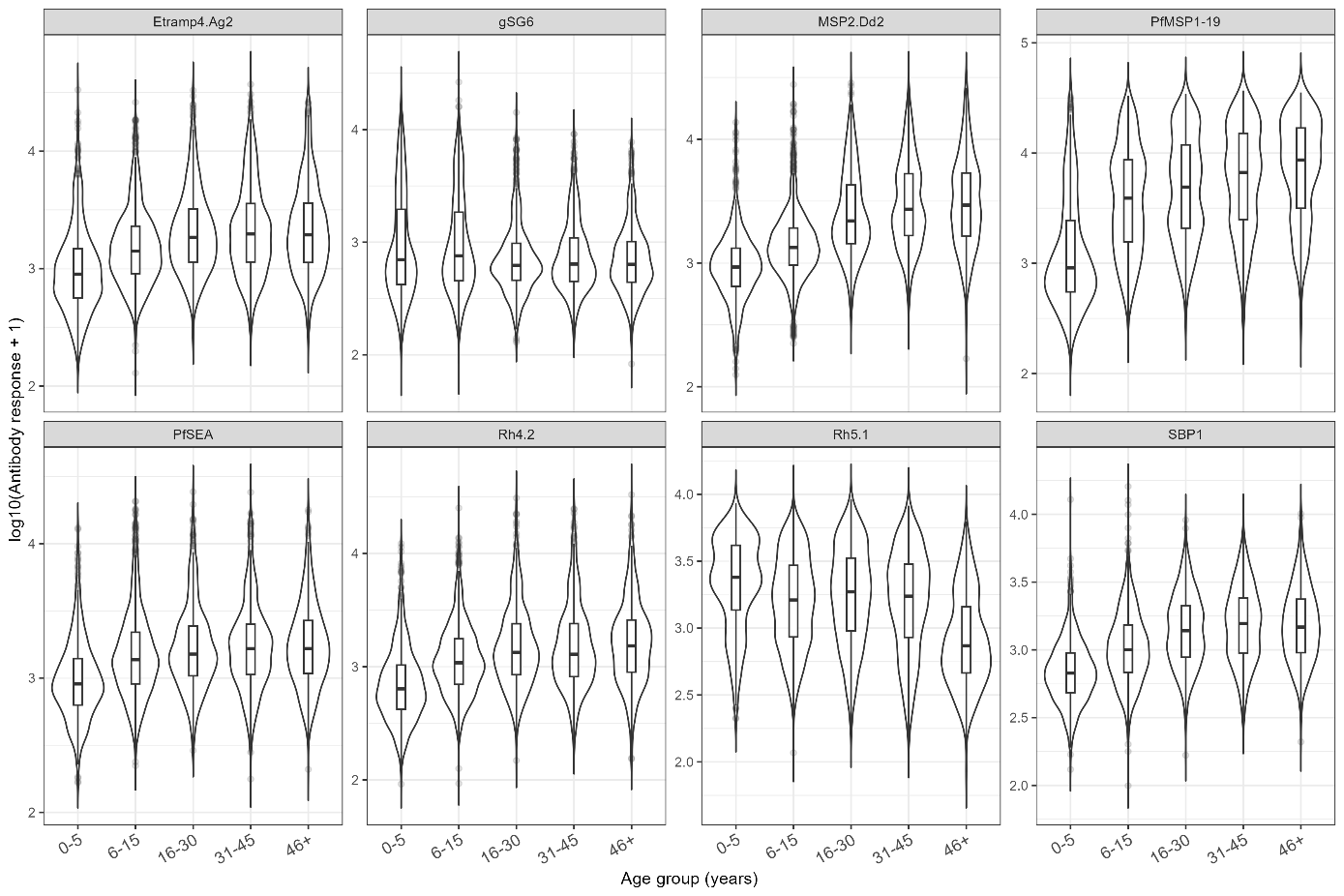
**

Violin plots showing the distribution of log10-transformed antibody responses (MFI + 1) across age groups among seropositive individuals in the <10% transmission stratum. Age was categorized into five groups (0–5, 6–15, 16–30, 31–45, and ≥46 years) for descriptive visualization. Each panel represents one of the eight antigens identified as top predictors in the random forest classification models. The violin shapes represent the density of antibody responses within each age group, while embedded boxplots indicate the median and interquartile range.

**Supplementary Figure 4.** Sex-specific distribution of antibody responses to top 8 predictive antigens

**
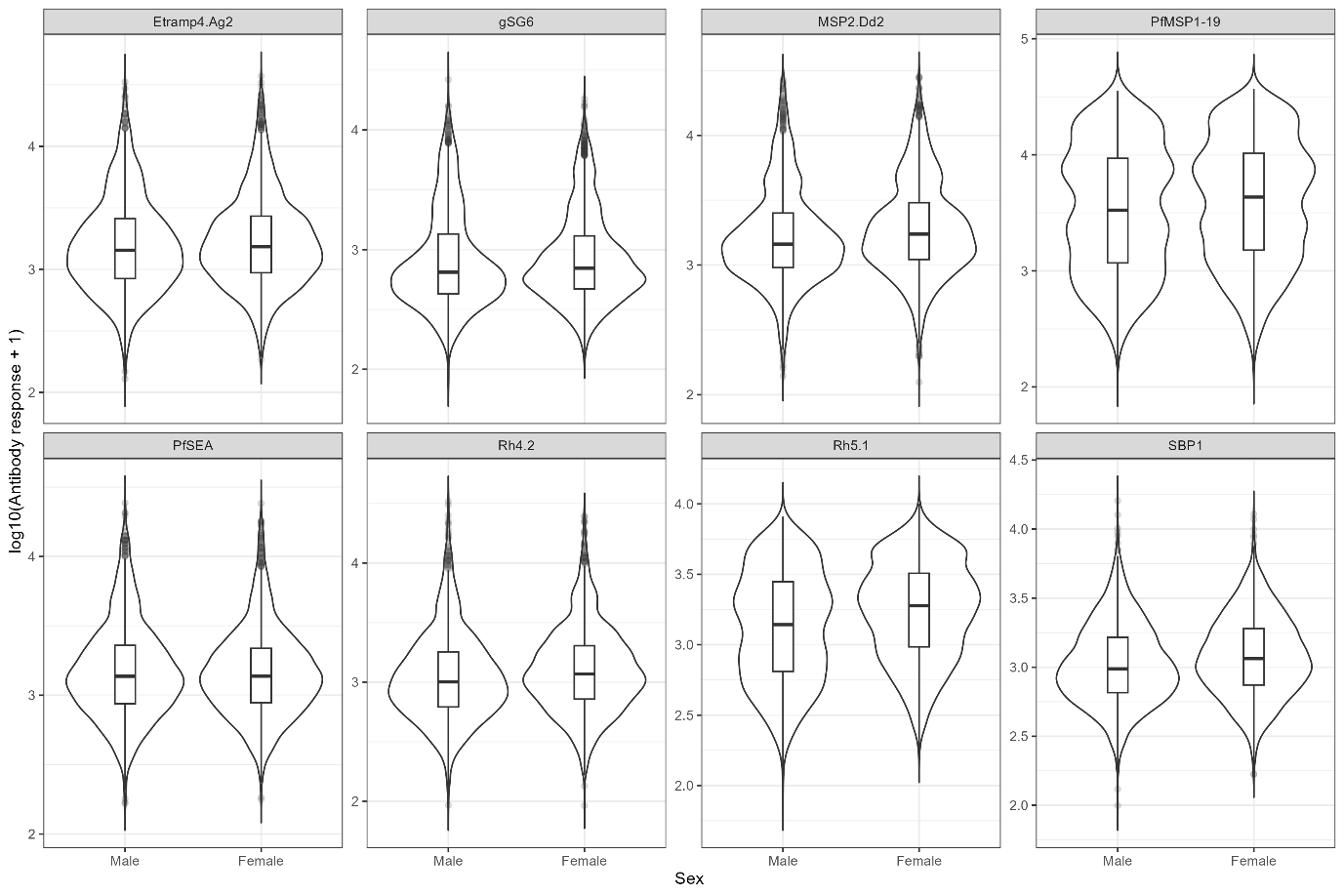
**

Violin plots showing the distribution of log10-transformed antibody responses (MFI + 1) by sex among seropositive individuals in the <10% transmission stratum. Each panel represents one of the eight antigens identified as top predictors in the random forest classification models. The violin shapes depict the density of antibody responses within each sex, while embedded boxplots indicate the median and interquartile range.

**Supplementary Figure 5.** Variable importance of antibody predictors from regression models across transmission settings among seropositive individuals.


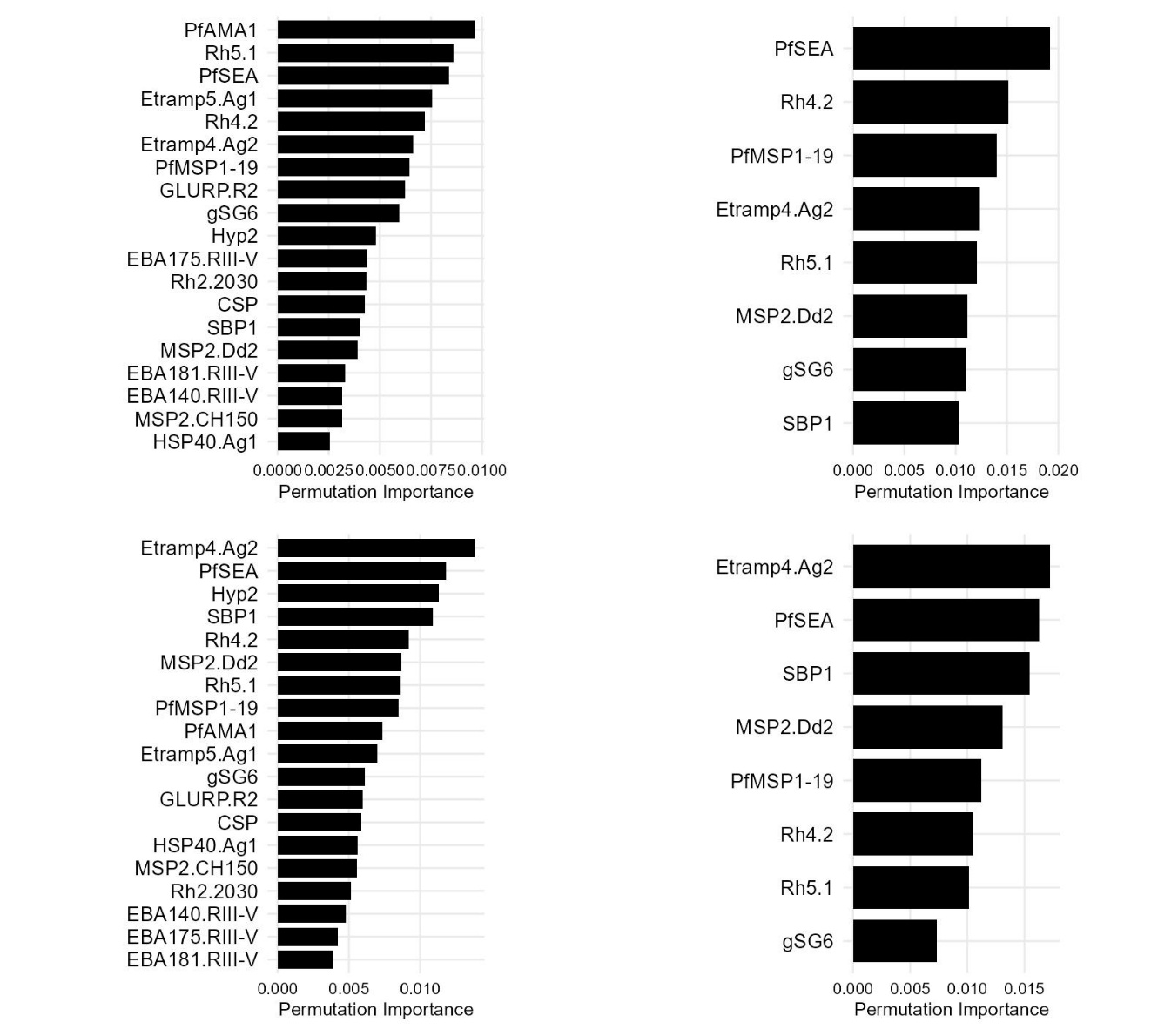


**(A)** <5% transmission, all 19 antigens; **(B)** <5% transmission, reduced 8 antigens. **(C)** <10% transmission, all 19 antigens. **(D)** <10% transmission, reduced 8 antigens. Higher importance values indicate greater influence on regression performance.

# Supplementary Tables

# Supplementary Table 1. Life-cycle stage and exposure classification of *Plasmodium falciparum* antigens included in the multiplex serological panel

| Antigen | Life-cycle stage | Exposure type |
| --- | --- | --- |
| Circumsporozoite Protein (CSP) | Sporozoite | Recent; cumulative (1, 2) |
| Early Transcribed Membrane Protein 5, Antigen 1 (Etramp5.Ag1) (4) | Erythrocytic blood (ring) | Recent (1, 3-5) |
| Early Transcribed Membrane Protein 4, Antigen 2 (Etramp4.Ag2) | Erythrocytic blood (ring) | Recent (3) |
| Apical Membrane Antigen 1 (PfAMA1) | Erythrocytic blood (merozoite) | Cumulative (1, 4) |
| Merozoite Surface Protein 1, 19 kDa fragment (PfMSP1-19) | Erythrocytic blood (merozoite) | Cumulative (1, 4) |
| Reticulocyte Binding Protein Homologue 5 (Rh5.1) | Erythrocytic blood (merozoite) | Cumulative (6, 7) |
| Reticulocyte Binding Protein Homologue 4  (Rh4.2) | Erythrocytic blood (merozoite) | Medium-term; insufficient evidence (1, 8) |
| Reticulocyte Binding Protein Homologue 2 (Rh2.2030) | Erythrocytic blood (merozoite) | Recent (4) (6) |
| Schizont Egress Antigen (PfSEA) | Erythrocytic blood (schizont) | Recent (9) (12) |
| Merozoite Surface Protein 2, Dd2 allele (MSP2.Dd2) | Erythrocytic blood (merozoite) | Cumulative (6, 10, 11) |
| Merozoite Surface Protein 2, CH150 allele (MSP2.CH150) | Erythrocytic blood (merozoite) | Cumulative (6, 11) |
| Skeleton Binding Protein 1 (SBP1) | Erythrocytic blood (ring/trophozoite) | Insufficient evidence (15, 16) |
| Hypothetical Protein 2 (Hyp2) | Erythrocytic blood (ring/trophozoite) | Recent (3, 11, 12) |
| Heat Shock Protein 40, Antigen 1 (HSP40.Ag1) | Erythrocytic blood | Recent (3, 4, 11) |
| Glutamate-Rich Protein, R2 region (GLURP.R2) | Erythrocytic blood (merozoite) | Cumulative (1, 2, 4, 13-17) |
| Erythrocyte Binding Antigen 175, regions III–V (EBA175.RIII-V) | Erythrocytic blood (merozoite) | Recent; cumulative (2, 4, 12, 18, 19) |
| Erythrocyte Binding Antigen 140, regions III–V (EBA140.RIII-V) | Erythrocytic blood (merozoite) | Cumulative (18-20) |
| Erythrocyte Binding Antigen 181, regions III–V (EBA181.RIII-V) | Erythrocytic blood (merozoite) | Cumulative (18, 19) |
| *Anopheles gambiae* Salivary Gland Protein 6 (gSG6) | Mosquito stage (saliva) | Recent (21) |

Note. Antibody kinetics vary substantially by transmission intensity, age, prior exposure history, and host immunological background. Antigens classified here as "cumulative" may still show some sensitivity to recent infection in low-transmission or naïve populations, and those classified as "recent" may persist longer in individuals with high cumulative exposure. PfSEA and SBP1 are flagged as having insufficient published kinetics evidence to support a confident classification in either direction. Publicly available sequences for all antigens are available at plasmodb.org.

# Supplementary Table 2. Performance metrics of random forest regression models predicting continuous P(Detect) across transmission settings in full study population.

| Transmission Level | Model Type | Antigen Set | RMSE | R^2^ |
| --- | --- | --- | --- | --- |
| <5% | Separate | All 19 | 0.15 | 0.11 |
|  |  | Reduced 8 | 0.15 | 0.15 |
|  | Combined mean MFI | All 19 | 0.15 | 0.02 |
|  |  | Reduced 8 | 0.16 | 0.02 |
| <10% | Separate | All 19 | 0.13 | 0.14 |
|  |  | Reduced 8 | 0.13 | 0.12 |
|  | Combined Mean MFI | All 19 | 0.14 | 0.01 |
|  |  | Reduced 8 | 0.14 | 0.01 |

# Supplementary Table 3. Performance metrics of random forest regression models predicting continuous P(Detect) across transmission settings among seropositive individuals.

| Transmission Level | Model Type | Antigen Set | RMSE | R^2^ |
| --- | --- | --- | --- | --- |
| <5% | Separate | All 19 | 0.15 | 0.09 |
|  |  | Reduced 8 | 0.14 | 0.12 |
|  | Combined mean MFI | All 19 | 0.15 | 0.02 |
|  |  | Reduced 8 | 0.15 | 0.03 |
| <10% | Separate | All 19 | 0.14 | 0.12 |
|  |  | Reduced 8 | 0.14 | 0.12 |
|  | Combined Mean MFI | All 19 | 0.15 | 0.008 |
|  |  | Reduced 8 | 0.15 | 0.003 |


# Supplementary Table 4. Median antibody levels (MFI-bg) in intervention and control arms in full study population.

| Antigen | Control: Median (IQR) | Intervention: Median (IQR) |
| --- | --- | --- |
| gSG6 | 654 (423–1271) | 644 (429–1216) |
| Etramp4.Ag2 | 1491 (833–2783) | 1398 (817–2629) |
| SBP1 | 999 (623–1805) | 1056 (685–1786) |
| Rh5.1 | 1598 (802–2946) | 1592 (846–2844) |
| Rh4.2 | 1024 (599–1855) | 1108 (664–2061) |
| PfSEA | 1346 (813–2316) | 1406 (907–2229) |
| MSP2.Dd2 | 1478 (922–2714) | 1551 (988–2830) |
| PfMSP1-19 | 3380 (1060–10502) | 3563 (1256–10334) |
| Etramp5.Ag1 | 847 (508–1654) | 847 (532–1534) |
| EBA181.RIII-V | 797 (504–1613) | 850 (554–1683) |
| Hyp2 | 1507 (1045–2192) | 1587 (1200–2166) |
| CSP | 558 (216–2194) | 655 (230–2596) |
| EBA140.RIII-V | 493 (237–1574) | 498 (243–1490) |
| EBA175.RIII-V | 421 (142–2113) | 458 (155–2521) |
| GLURP.R2 | 1400 (199–11815) | 1797 (232–12045) |
| HSP40.Ag1 | 1380 (862–2391) | 1382 (953–2274) |
| MSP2.CH150 | 1144 (328–5024) | 1249 (342–5515) |
| PfAMA1 | 3384 (426–14494) | 4608 (484–14593) |
| Rh2.2030 | 1584 (779–5559) | 1634 (820–5578) |

# Supplementary Table 5. Performance metrics of random forest classification models predicting low versus high P(Detect) across transmission settings in full study population.

| Transmission Level | Model Type | Antigen Set | ROC AUC | PR AUC | Accuracy |
| --- | --- | --- | --- | --- | --- |
| <5% | Separate | All 19 | 0.75 | 0.94 | 0.83 |
|  |  | Reduced 8 | 0.77 | 0.94 | 0.83 |
|  | Combined mean MFI | All 19 | 0.58 | 0.86 | 0.82 |
|  |  | Reduced 8 | 0.59 | 0.88 | 0.83 |
| <10% | Separate | All 19 | 0.78 | 0.91 | 0.76 |
|  |  | Reduced 8 | 0.76 | 0.91 | 0.76 |
|  | Combined Mean MFI | All 19 | 0.58 | 0.81 | 0.74 |
|  |  | Reduced 8 | 0.58 | 0.79 | 0.74 |

# Supplementary Table 6. Linear regression analysis of standardized antibody responses associated with continuous P(Detect), with and without adjustment for age and sex.

| Antigen | Unadjusted β (95% CI) | p-value | Adjusted β (95% CI) | p-value |
| --- | --- | --- | --- | --- |
| gSG6 | 0.012 (0.007, 0.016) | <0.001 | 0.012 (0.008, 0.017) | <0.001 |
| Etramp4.Ag2 | 0.009 (0.005, 0.014) | <0.001 | 0.009 (0.005, 0.014) | <0.001 |
| SBP1 | 0.001 (-0.004, 0.006) | 0.661 | 0.000 (-0.005, 0.005) | 0.910 |
| Rh5.1 | 0.020 (0.015, 0.024) | <0.001 | 0.021 (0.016, 0.026) | <0.001 |
| Rh4.2 | 0.003 (-0.002, 0.008) | 0.190 | 0.003 (-0.002, 0.007) | 0.246 |
| PfSEA | -0.002 (-0.007, 0.002) | 0.288 | -0.003 (-0.007, 0.002) | 0.287 |
| MSP2.Dd2 | -0.009 (-0.014, -0.005) | <0.001 | -0.011 (-0.016, -0.006) | <0.001 |
| PfMSP1-19 | -0.003 (-0.008, 0.002) | 0.188 | -0.004 (-0.009, 0.001) | 0.094 |

Linear regression models examining associations between standardized antibody responses (per 1 standard deviation increase in antibody level) and continuous P(Detect). Unadjusted models include each antigen predictor individually. Adjusted models additionally include age (continuous) and sex as covariates. Beta coefficients represent the change in P(Detect) associated with a one standard deviation increase in antibody response. Highlighted cells represent significant values.

**References**

1. Carcelen AC, Monjane C, Bérubé S, Takahashi S, Sultane T, Chelene I, et al. Multiplex bead assays enable integrated serological surveillance and reveal cross-pathogen vulnerabilities in Zambezia Province, Mozambique. Nature Communications. 2025;16(1):7946.

2. Ondigo BN, Hamre KES, Frosch AEP, Ayodo G, White MT, John CC. Antibody Profiles to P. falciparum Antigens Over Time Characterize Acute and Long-Term Malaria Exposure in an Area of Low and Unstable Transmission. Am J Trop Med Hyg. 2020;103(6):2189-97.

3. Helb DA, Tetteh KK, Felgner PL, Skinner J, Hubbard A, Arinaitwe E, et al. Novel serologic biomarkers provide accurate estimates of recent Plasmodium falciparum exposure for individuals and communities. Proc Natl Acad Sci U S A. 2015;112(32):E4438-47.

4. Wu L, Mwesigwa J, Affara M, Bah M, Correa S, Hall T, et al. Sero-epidemiological evaluation of malaria transmission in The Gambia before and after mass drug administration. BMC Med. 2020;18(1):331.

5. Druetz T, van den Hoogen L, Stresman G, Joseph V, Hamre KES, Fayette C, et al. Etramp5 as a useful serological marker in children to assess the immediate effects of mass drug campaigns for malaria. BMC Infect Dis. 2022;22(1):643.

6. Yman V, White MT, Asghar M, Sundling C, Sondén K, Draper SJ, et al. Antibody responses to merozoite antigens after natural Plasmodium falciparum infection: kinetics and longevity in absence of re-exposure. BMC Med. 2019;17(1):22.

7. Natama HM, Salkeld J, Somé A, Soremekun S, Diallo S, Traoré O, et al. Safety and efficacy of the blood-stage malaria vaccine RH5.1/Matrix-M in Burkina Faso: interim results of a double-blind, randomised, controlled, phase 2b trial in children. The Lancet Infectious Diseases. 2025;25(5):495-506.

8. Reiling L, Richards JS, Fowkes FJ, Wilson DW, Chokejindachai W, Barry AE, et al. The Plasmodium falciparum erythrocyte invasion ligand Pfrh4 as a target of functional and protective human antibodies against malaria. PLoS One. 2012;7(9):e45253.

9. Yman V, Tuju J, White MT, Kamuyu G, Mwai K, Kibinge N, et al. Distinct kinetics of antibodies to 111 Plasmodium falciparum proteins identifies markers of recent malaria exposure. Nat Commun. 2022;13(1):331.

10. Feng G, Boyle MJ, Cross N, Chan JA, Reiling L, Osier F, et al. Human Immunization With a Polymorphic Malaria Vaccine Candidate Induced Antibodies to Conserved Epitopes That Promote Functional Antibodies to Multiple Parasite Strains. J Infect Dis. 2018;218(1):35-43.

11. Ezinmegnon S, Nébié I, Marlais T, Ouattara D, Diarra A, Patterson C, et al. A Multiplex Serological Assay to Evaluate the Antibody Responses to a Set of Plasmodium falciparum Antigens and Their Protective Role Against Malaria in Children Aged 1.5 to 12 Years Living in a Highly Seasonal Malaria Transmission Area of Burkina Faso. Vaccines. 2025;13(11):1091.

12. van den Hoogen LL, Stresman G, Présumé J, Romilus I, Mondélus G, Elismé T, et al. Selection of Antibody Responses Associated With Plasmodium falciparum Infections in the Context of Malaria Elimination. Front Immunol. 2020;11:928.

13. Ondigo BN, Hodges JS, Ireland KF, Magak NwG, Lanar DE, Dutta S, et al. Estimation of Recent and Long-Term Malaria Transmission in a Population by Antibody Testing to Multiple Plasmodium falciparum Antigens. The Journal of Infectious Diseases. 2014;210(7):1123-32.

14. Kana IH, Singh SK, Garcia-Senosiain A, Dodoo D, Singh S, Adu B, et al. Breadth of Functional Antibodies Is Associated With Plasmodium falciparum Merozoite Phagocytosis and Protection Against Febrile Malaria. The Journal of Infectious Diseases. 2019;220(2):275-84.

15. Kerkhof K, Sluydts V, Willen L, Kim S, Canier L, Heng S, et al. Serological markers to measure recent changes in malaria at population level in Cambodia. Malaria Journal. 2016;15(1):529.

16. Macalinao MLM, Fornace KM, Reyes RA, Hall T, Bareng APN, Adams JH, et al. Analytical approaches for antimalarial antibody responses to confirm historical and recent malaria transmission: an example from the Philippines. The Lancet Regional Health – Western Pacific. 2023;37.

17. Drakeley CJ, Corran PH, Coleman PG, Tongren JE, McDonald SLR, Carneiro I, et al. Estimating medium- and long-term trends in malaria transmission by using serological markers of malaria exposure. Proceedings of the National Academy of Sciences. 2005;102(14):5108-13.

18. McCallum FJ, Persson KEM, Fowkes FJI, Reiling L, Mugyenyi CK, Richards JS, et al. Differing rates of antibody acquisition to merozoite antigens in malaria: implications for immunity and surveillance. Journal of Leukocyte Biology. 2016;101(4):913-25.

19. Richards JS, Stanisic DI, Fowkes FJI, Tavul L, Dabod E, Thompson JK, et al. Association between Naturally Acquired Antibodies to Erythrocyte-Binding Antigens of Plasmodium falciparum and Protection from Malaria and High-Density Parasitemia. Clinical Infectious Diseases. 2010;51(8):e50-e60.

20. Maier AG, Duraisingh MT, Reeder JC, Patel SS, Kazura JW, Zimmerman PA, et al. Plasmodium falciparum erythrocyte invasion through glycophorin C and selection for Gerbich negativity in human populations. Nat Med. 2003;9(1):87-92.

21. Ndo C, Elanga-Ndille E, Cheteug G, Metitsi RD, Wanji S, Moukoko CEE. IgG antibody responses to Anopheles gambiae gSG6-P1 salivary peptide are induced in human populations exposed to secondary malaria vectors in forest areas in Cameroon. PLoS One. 2022;17(11):e0276991.
